# A Case Report of Unilateral Hypertrophy of Foot Intrinsics: Elastographic Properties of Hyperthrophied Muscles

**DOI:** 10.7759/cureus.25724

**Published:** 2022-06-07

**Authors:** Sena Tuncer, Yahya Deniz, Onur Yildirim, Gokhan Kaynak, Bedri Karaismailoglu

**Affiliations:** 1 Orthopaedics and Traumatology, Istanbul University-Cerrahpasa, Istanbul, TUR

**Keywords:** shear wave elastography, surgical excision, congenital, foot intrinsics, muscle hypertrophy

## Abstract

Congenital hypertrophy of intrinsic foot muscles is a rare condition. We report an unusual case of a 24-year-old male with a painless swelling at the plantar and medial aspect of the right foot, which was present since birth. The significant size of deformity and discomfort in wearing shoes were major concerns. MRI revealed expansion of multiple intrinsic foot muscles, which are abductor hallucis, flexor digitorum brevis, and quadratus plantae. We used shear wave elastography (SWE) as an imaging technique besides MRI and ultrasonography, which has not been used or published previously. Muscle shear wave velocity values were measured in abductor hallucis, flexor hallucis brevis, flexor digitorum brevis, and quadratus plantaris muscles. The mean stiffness values of muscles on the affected side were significantly higher compared to the healthy side. The median SWE value was 6.67 kPa (range: 4.7-8.6 kPa) on the healthy side, while it ranged between 9.2 and 13.4 kPa on the affected side. Total excision of abductor hallucis and subtotal resection of flexor digitorum brevis and quadratus plantae muscles were performed, and motor and sensory functions were preserved by protecting the neurovascular bundle. Excess skin was also removed. Surgery and postoperative course were uneventful. The patient was allowed to bear weight as tolerated. There was no recurrence, and the patient was satisfied with the shape and size of his foot at the six-month follow-up. Congenital hypertrophy of foot muscle is uncommon especially when multiple intrinsic foot muscles are involved. The main aim is to relieve patients’ concerns, correct deformities, and provide comfortable shoe wear.

## Introduction

Congenital hypertrophy of the intrinsic foot muscles is rare. The first available report was published by Jahss in 1974, and he reported hypertrophy of the quadratus plantaris muscle [1]. Only few cases have been reported in the literature since then, and multiple involvement of foot intrinsics is also less common [2]. We report an unusual case of a 24-year-old male with a painless swelling at the plantar aspect of the right foot, which was present since birth. We also used shear wave elastography (SWE) as an imaging modality, which has not been used or published previously. SWE provides the measurement of tissue stiffness, which can change due to underlying pathology, and also allows comparing the values with the healthy side.

## Case presentation

**Figure 1:**
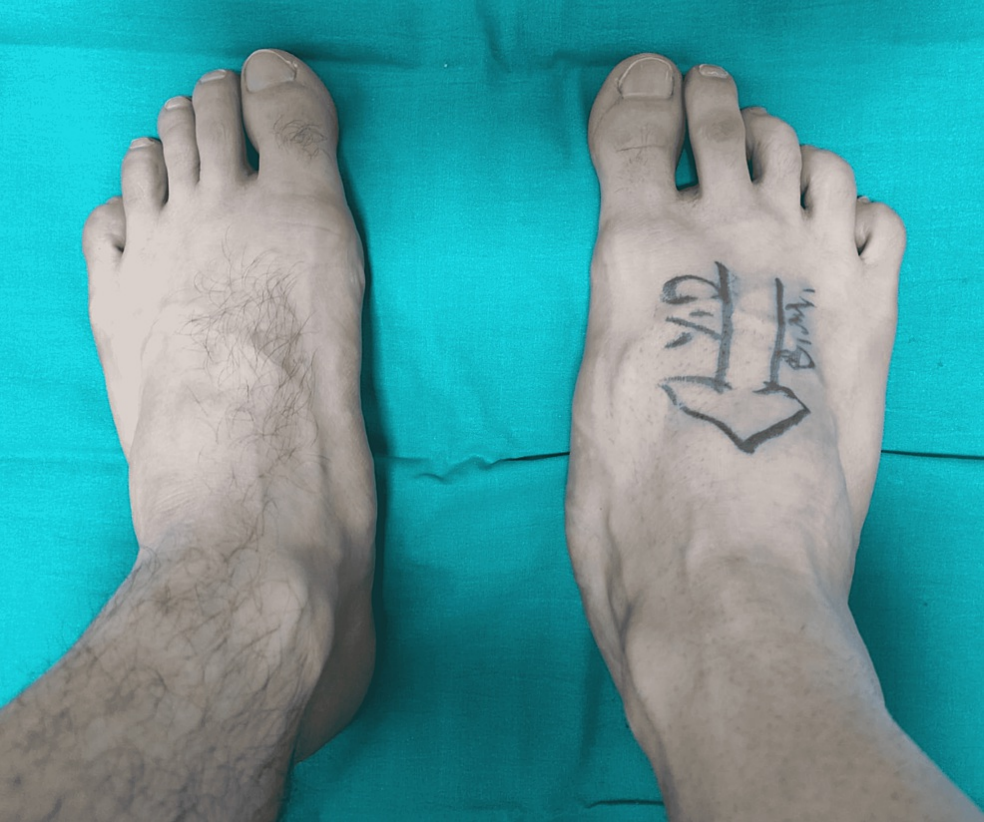
First presentation of the patient. A mass can be seen on the medial side of the right foot.

A 24-year-old healthy male with painless swelling involving the plantar and medial aspect of his right foot presented to our department (Figure 1).

The mass was present since his birth. His birth was uneventful, and he had no history of congenital anomalies or connective tissue diseases. The clinical examination revealed a soft tissue tumor at the plantar and medial aspect of his right foot without tenderness. There were no abnormalities in the motor and sensory function, and the skin was normal. The significant size of deformity and discomfort in shoe wear were the major concerns.

**Figure 2:**
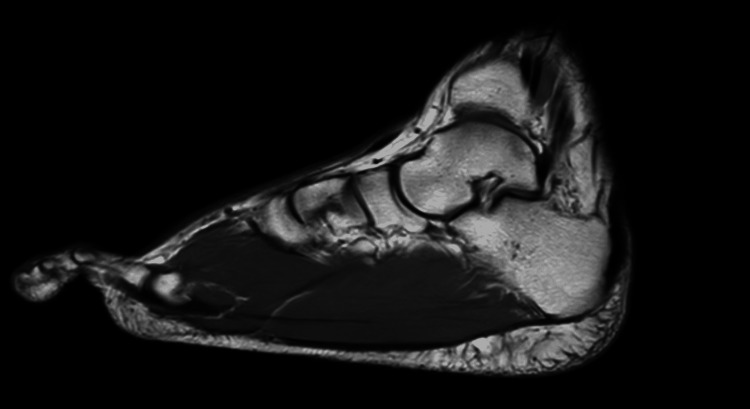
Preoperative MRI showing hypertrophy of multiple intrinsic foot muscles.

X-rays showed enlargement of soft tissue with unaffected bone structure. Ultrasonography of the right foot showed hypertrophic muscles with normal architecture of surrounding tissue. Magnetic resonance imaging (MRI) revealed expansion of multiple intrinsic foot muscles, which included the abductor hallucis, flexor digitorum brevis, and quadratus plantae muscles (Figure 2).

Muscle shear wave velocity values were measured for abductor hallucis, flexor hallucis brevis, flexor digitorum brevis, and quadratus plantaris muscles in both sides. The mean stiffness values of muscles on the affected side were significantly higher compared to that of the contralateral side. The median SWE value was 6.67 kPa (range: 4.7-8.6 kPa) on the healthy side, while the affected foot had higher values, which ranged between 9.2 and 13.4 kPa (Table 1).

**Table 1:** Shear wave elastography values for affected and unaffected sides.

Muscle	Unaffected Foot	Affected Foot
Abductor hallucis	6.5 kPa	11.6 kPa
Flexor hallucis brevis	8.6 kPa	9.2 kPa
Flexor digitorum brevis	4.7 kPa	9.5 kPa
Quadratus plantaris	6.9 kPa	13.4 kPa

**Figure 3:**
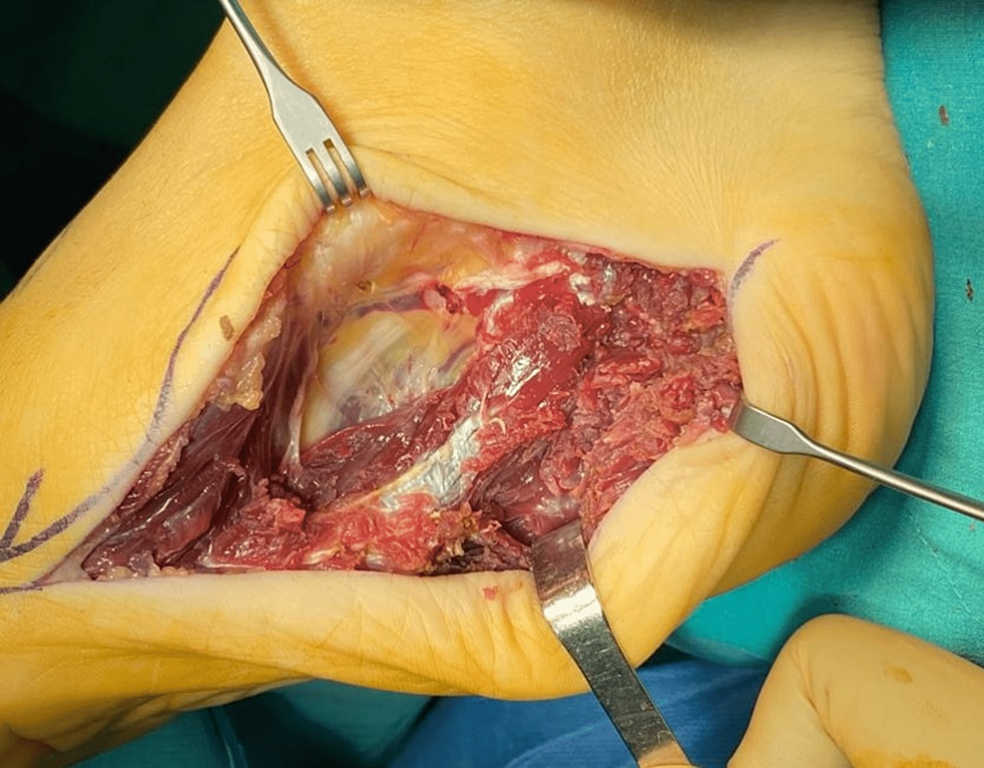
Intraoperative photo displaying the medial approach and neurovascular bundle superiorly.

Total excision of the abductor hallucis and subtotal resection of the flexor digitorum brevis and quadratus plantae muscles were planned. Adjacent neurovascular bundle was protected to preserve motor and sensory function (Figure 3). 

**Figure 4:**
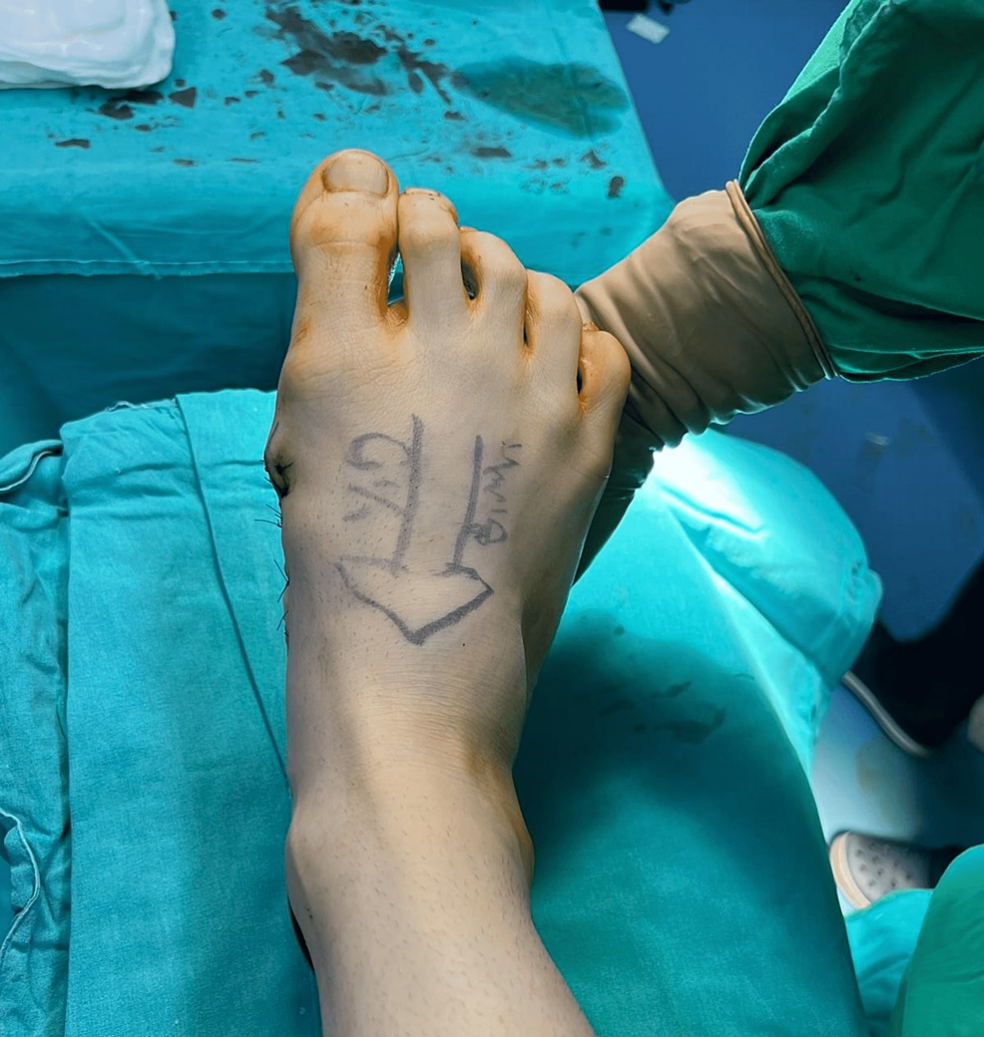
Intraoperative photograph after skin closure showing appropriate foot size for comfortable shoe wear.

Excess skin was also removed during skin closure. No complication occurred, and an appropriate shape of the foot was achieved after skin closure (Figure 4).

**Figure 5:**
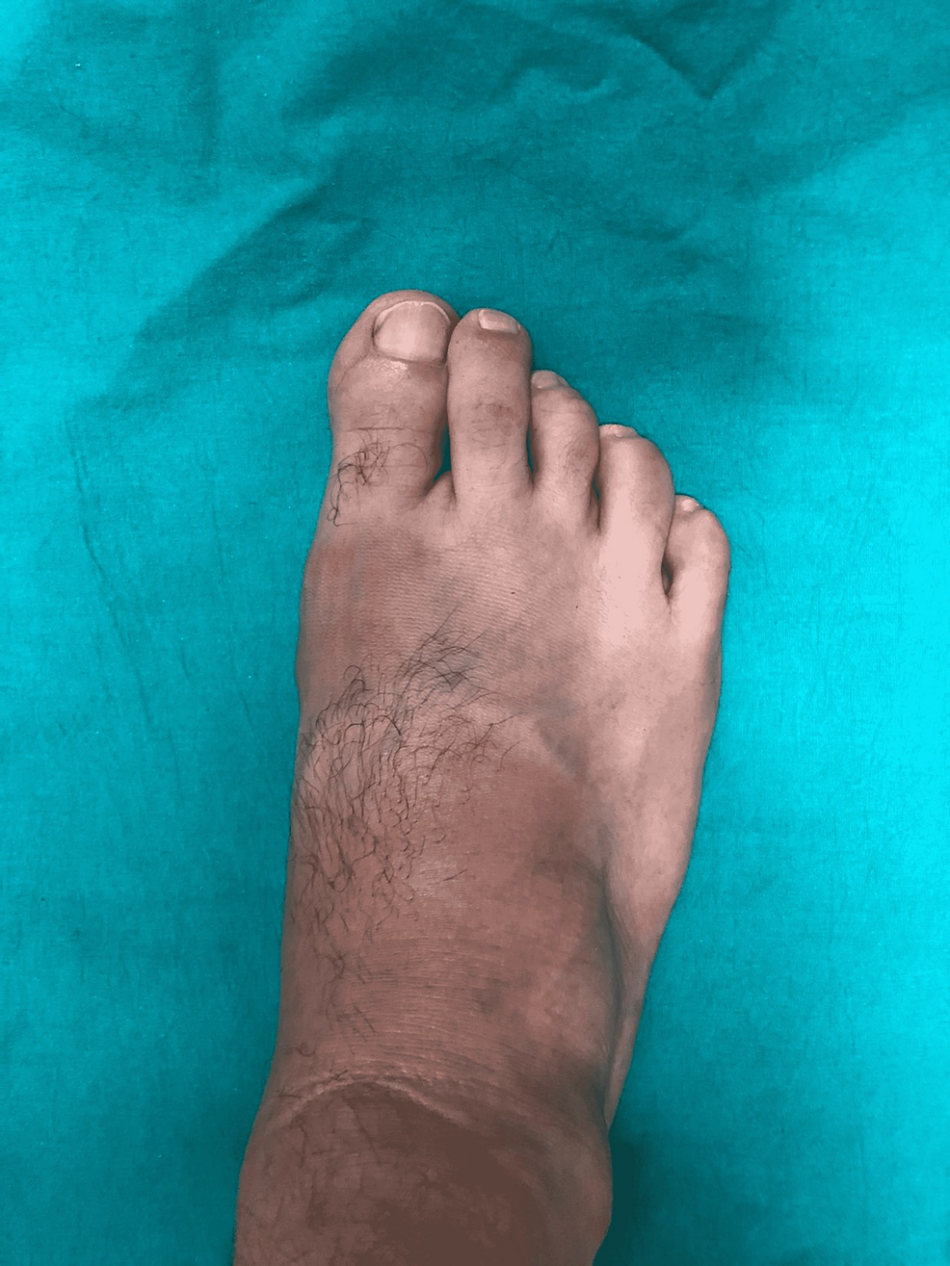
Photograph from the six-month follow-up. The patient was satisfied with his foot shape and was able to wear shoes comfortably.

The patient was allowed to bear weight as tolerated. No recurrence was present, and the patient was satisfied with the shape and size of his foot, and he was able to wear his shoes comfortably (Figure 5).

## Discussion

Congenital hypertrophy of intrinsic foot muscles is a rare condition. Only few reports have been reported until today, and multiple muscle involvement is less commonly reported [2]. Although these cases have some common characteristics such as they are all soft tissue masses present from birth, unilateral, non-tender to palpation, and generally non-progressive, and they all show normal bony anatomy on radiographs [3], there is no apparent common etiology or known incidence [1]. Age of the patients at the time of intervention range between 4 months and 33 years. Patients with this disorder usually exhibit foot asymmetry, and their major concern is uncomfortable shoe wear. In athletic children and adolescents, hypertrophy may be a result of abundant exercise [4]. However, our patient did not report excess physical activity in his past. Furthermore, hypertrophy of bone and soft tissue with associated lipomatosis [5] and the development of benign and malign tumors should also be considered among the differential diagnoses [6].

The first case of hypertrophy of multiple intrinsic foot muscles was reported in 2013 by Shiraishi et al. [2]. The patient was a four-month-old boy who presented with asymmetrical growth in the left foot. Radiologic images showed that the size and shape of bones in both feet were same. MRI confirmed hypertrophy of intrinsic foot muscles as suspected. When the patient became one-year-old, the affected muscles including the abductor digiti minimi, flexor digiti minimi, abductor hallucis, medial head of the flexor hallucis brevis, and flexor digitorum brevis muscles were partially excised. Surgery and postoperative course were uneventful. However, volumetric difference between the two feet slightly remained at the one-year follow-up.

The case reported by Holzbauer et al. in 2020 involved a 17-year-old man with hypertrophy of the right foot [7]. The patient developed multiple toe deformities such as pes planus with hallux valgus deformity, claw deformity on the second toe and digitus quintus varus. MRI showed subcutaneous fat hypertrophy beside muscular hypertrophy, unlike the case we present. In surgery, hypertrophic abductor hallucis brevis and flexor digitorum brevis muscles were resected, and hallux valgus deformity was treated by plication of the medial part of the capsule and proximal osteotomy of the first metatarsal.

In our study, SWE was also performed to show elastographic difference of the pathological muscles. The values revealed that hypertrophied muscles were stiffer with less elasticity, probably due to non-functioning nature of the muscles without any basal activity.

## Conclusions

Congenital hypertrophy of foot muscles is an uncommon disorder, especially if it involves multiple intrinsic foot muscles. It tends to not involve surrounding tissues. The main aims are relieving patients’ concerns, correcting the deformities, and preventing recurrence of the problems.
